# Effectiveness of Propionyl-L-Carnitine Supplementation on Exercise Performance in Intermittent Claudication: A Systematic Review

**DOI:** 10.7759/cureus.17592

**Published:** 2021-08-31

**Authors:** Belen Tama, Stephanie P Fabara, Daniel Zarrate, Anas Anas Sohail

**Affiliations:** 1 Medicine, Universidad Católica de Santiago de Guayaquil, Guayaquil, ECU; 2 Internal Medicine, Universidad Católica de Santiago de Guayaquil, Guayaquil, ECU; 3 General Medicine, El Bosque University, Bogotá, COL; 4 Medicine, Aureus University School of Medicine, Oranjestad, ABW

**Keywords:** intermittent claudication, peripheral artery disease, propionyl-l-carnitine, l-carnitine, exercise performance, peak walking time

## Abstract

Lower extremity peripheral artery disease (PAD) affects 8.5 million people in the United States and more than 200 million worldwide. The most significant risk factors for PAD are hyperlipidemia, hypertension, diabetes mellitus, chronic kidney disease, and smoking. Intermittent claudication (IC) is the predominant symptom of PAD, but only about 10% of patients with PAD experience IC and are associated with reduced exercise capacity. The pathophysiology of IC is characterized by different degrees of stenosis and obstruction, with a progressive reduction in distal perfusion pressure and blood flow. Supervised exercise therapy is recommended as the initial therapy for IC, but the recommendations for medical treatment of IC vary significantly. Propionyl L-carnitine is an acyl derivative of levocarnitine (L-carnitine) and is indicated for patients with the peripheral arterial occlusive disease. It corrects secondary muscle carnitine deficiency in patients with PAD, significantly improving the walking capacity; its levels increase in serum and muscle. Thus, it is suggested to enhance blood flow and oxygen supply to the muscle tissue via improved endothelial function, thereby reducing hypoxia-induced cellular and biochemical disruptions.

## Introduction and background

Peripheral artery disease (PAD) of the lower limbs is one manifestation of atherosclerotic vascular disease, affecting 3%-7% of the general population and 20% of individuals over 75 years [1]. Despite its high prevalence, PAD often remains unrecognized and underdiagnosed [2]. PAD development is multifactorial, with a complex interplay of modifiable and non-modifiable risk factors. Cigarette smoking is one of the strongest risk factors, with over half of patients with PAD being smokers [1]. Diabetes is also strongly associated with PAD, with the strongest associations seen in patients with long-standing and uncontrolled diabetes [2]. An ankle-brachial index of ≤0.90 is generally considered diagnostic of PAD for both epidemiological and clinical purposes [2]. Intermittent claudication (IC) is the main symptom of PAD, defined as a very limiting cramping leg pain (in the buttock, thigh, or calf) that can markedly reduce walking speed, walking distance, and stair-climbing ability. It is also associated with difficulty walking indoors and walking distance limited to typically 100-200 meters (1-2 blocks) [3].

Pathophysiologically, the pain results from the discrepancy between oxygen demand and arterial supply. IC affects about 12% of adults and 20% of individuals over 70 years of age [4]. In the exercising muscle, calf muscle perfusion fails to sufficiently increase blood flow to match metabolic demands, with ensuing muscle ischemia. This vicious cycle of exercise-induced ischemia followed by reperfusion leads to the formation of reactive oxygen species that would subsequently cause an abnormal myocyte metabolism and finally, an impaired contractile performance [2]. Physical training is universally recognized as the most efficacious tool for improving the walking capacity in patients with PAD and should always accompany pharmacological treatment [4]. There are several pharmacologic approaches to the treatment of claudication, including drugs that have metabolic actions such as carnitine, an essential cofactor for skeletal muscle metabolism [5]. There is evidence that L-carnitine alleviates muscle injury and reduces markers of cellular damage and free radical formation accompanied by attenuation of muscle soreness [6]. Propionyl-L-carnitine (PLC) is an acyl derivative of levocarnitine (L-carnitine). L-carnitine is an endogenous quaternary amine that is ingested via animal-based food products or synthesized endogenously from the essential amino acids - methionine and lysine - in the kidney, liver, and brain [6].

It is estimated that dietary intake accounts for 75% of the body’s carnitine reservoir, red meat being the primary source. In contrast, vegetarians obtain very little L-carnitine from dietary sources because plant-derived foods contain insignificant quantities of L-carnitine [6]. Under normal metabolic conditions, skeletal and myocardial cells derive energy from the mitochondrial oxidation of fatty acids. L-carnitine is imperative for transporting fatty acids across the mitochondrial membrane for oxidation and energy production [7]. Due to the impermeability of the mitochondrial membrane to coenzyme A (CoA) and long-chain fatty acids, binding of L-carnitine to acetyl groups via carnitine acyltransferase is necessary for the shuttle of the acetylated fatty acids into the mitochondria and for their subsequent β-oxidation in the matrix. The products of the β-oxidation are then used by the Krebs cycle to produce adenosine triphosphate (ATP) as a form of energy [6]. It has been reported that the decreased level of carnitine in muscle is positively related to the decrease in the patients' maximum walking distance [7]. However, there is limited literature regarding PLC efficacy in patients with PAD. We designed this study to investigate propionyl L-carnitine’s efficacy, safety, and tolerability in patients with IC.

## Review

Materials and Methods

Protocol

For this systematic review, we used the Preferred Reporting Items for Systematic Reviews and Meta-Analysis (PRISMA) for clinical trials.

Eligibility Criteria and Study Selection

We only included clinical trials on humans in the last 20 years in the English literature. We excluded all animal studies, studies other than clinical trials, and those that did not fulfill the study's outcome. Patients in the study must have IC. After this process, we removed duplicate papers and studies in which the title was not pertinent.

After screening the studies, we included papers with the following criteria:

1. Patients: Individuals with PAD with IC symptoms

2. Intervention: PLC supplementation on exercise performance

3. Comparator: Treatment or control group

4. Outcome: Improvement of peak walking time

Database and Search Strategy

An electronic search of the literature using PubMed was performed from July to August 2021. The combination of search terms used was "L-carnitine'' and "claudication". We used an advanced search strategy with the following terms: (L-carnitine [Title/Abstract]) AND (claudication [Title/Abstract]).

Data Extraction and Analysis

We collected the following information for each study: author and year of publication, methodology, and functional outcomes. Baseline characteristics of the study methods include the number of participants in the treatment; the number of participants in the control group; and the dose, duration, and route of administration of the drugs. The primary efficacy endpoint was the improvement of peak walking time (PWT) after treatment over baseline on a graded treadmill protocol.

Bias Assessment

For assessing bias, we used the Cochrane collaboration's tool risk assessment [8] of the clinical trials.

Results

**Figure 1:**
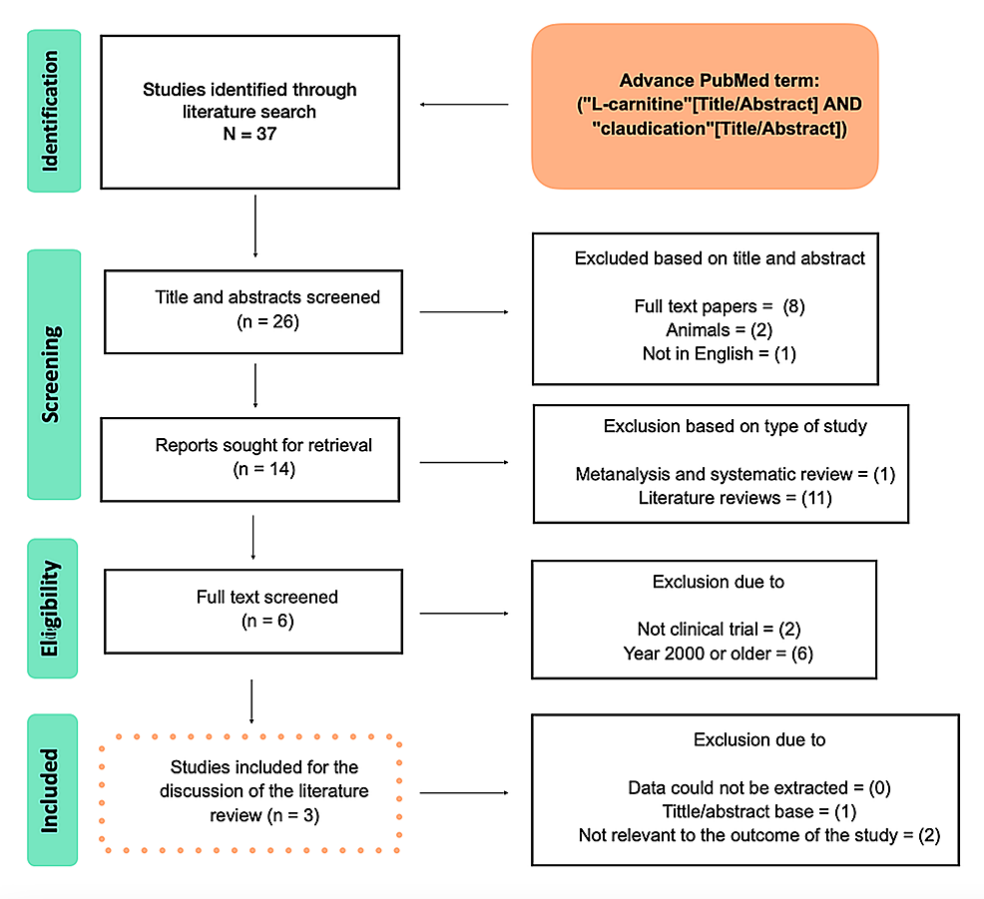
Results of the study using a PRISMA flow chart PRISMA, Preferred Reporting Items for Systematic Reviews and Meta-Analysis.

Figure 1 shows the results of the study using a PRISMA flow chart.

In total, three randomized clinical trials (RCT) were contemplated to be eligible for this systematic review. All of them had a control group, baseline characteristics, and a final measurable outcome.

Table 1 shows the author, year, country, study design, number of patients in treatment and control group, dose, duration, route of drug administration, and outcomes [5,7,9].

**Table 1:** Systematic review of the three randomized clinical trials PLC, Propionyl-L-carnitine; PWT, peak walking time.

Author, year of the publication	Country	Study design	Number of patients in the treatment group	Number of patients in the control group	Dose, duration, and route	Outcomes
Hiatt et al., 2001 [5]	USA	Double-blind, randomized, placebo-controlled trial	82	73	PLC 2 g/day orally or placebo for 6 months	Patients with PLC treatment increased their PWT by 162 ± 222 seconds (a 54% increase) in contrast to placebo that had an improvement of 75 ± 191 seconds (a 25% increase) (P = 0.001).
Luo et al., 2013 [7]	China	Randomized, multicenter, phase III, double-blind, parallel-group study	120	119	PLC 2 g/day orally or placebo for 4 months	PWT of the intervention group increased 1.6 ± 1.6 minutes after treatment (P = 0.05).
Hiatt et al., 2011 [9]	USA	Randomized, placebo-controlled, double-blinded, multicenter trial	32	30	PLC 2 g/day orally or placebo for 6 months	Patients with training and placebo had a PWT increase of 218 ± 367 seconds, while those with training and PLC had a 266 ± 243 seconds increase (P = 0.285).

Limitations of the Clinical Trials

Limitations in Hiatt et al.’s study were differences that could not be justified by protocol violations or unblinding of study medication, as ruled by a post-study audit by the sponsor [5]. These subjects were from two different countries, Russia and United States, with several between-country differences. At enrollment, Russian subjects had a higher PWT suggesting less severe claudication compared to those from the United States. Despite these differences, when the country was included in the prespecified analytic model, the treatment effect remained significant indicating that benefits of PLC were observed in both countries [5].

Luo et al.'s limitations were mainly attributable to the design of the patient visit protocol, suggesting that more visits should have been done in order to have comprehensive data [7].

**Figure 2:**
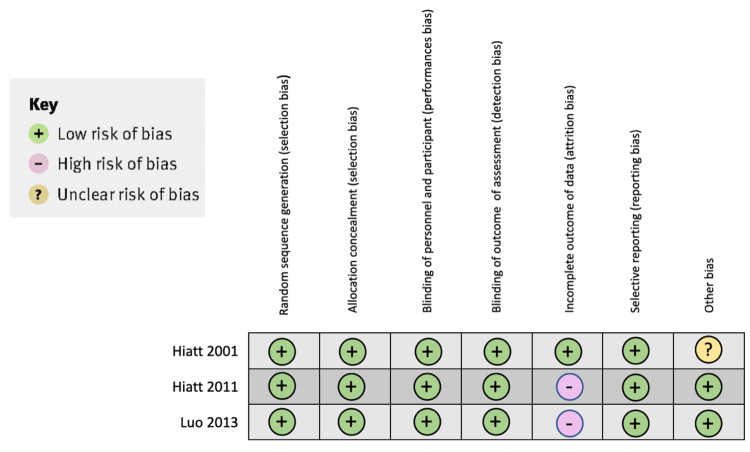
The bias analysis of the clinical trials in the systematic review

In Hiatt et al.'s study, due to the limited sample size, there was a lack of statistical significance. The background exercise addition could have influenced the results based on individual responses. An apparent imbalance between groups occurred in the distribution of women and in persons with diabetes, which also may reflect the small sample size. Monitored home exercise may be a helpful modality for prescribing exercise in patients with claudication [9]. Figure 2 shows the bias analysis of the clinical trials in the systematic review.

Discussion

For the present study, we analyzed IC, the main symptom of PAD, defined as cramping leg pain (in the buttock, thigh, or calf) while performing a minimal or considerable amount of exercise such as climbing one or two flights of stairs or during walking; meanwhile, other symptoms (e.g., non-healing wounds, ulceration, gangrene, and threatened limb) and combination treatment with other drugs were not analyzed but discussed because they were not of our interest.

**Figure 3:**
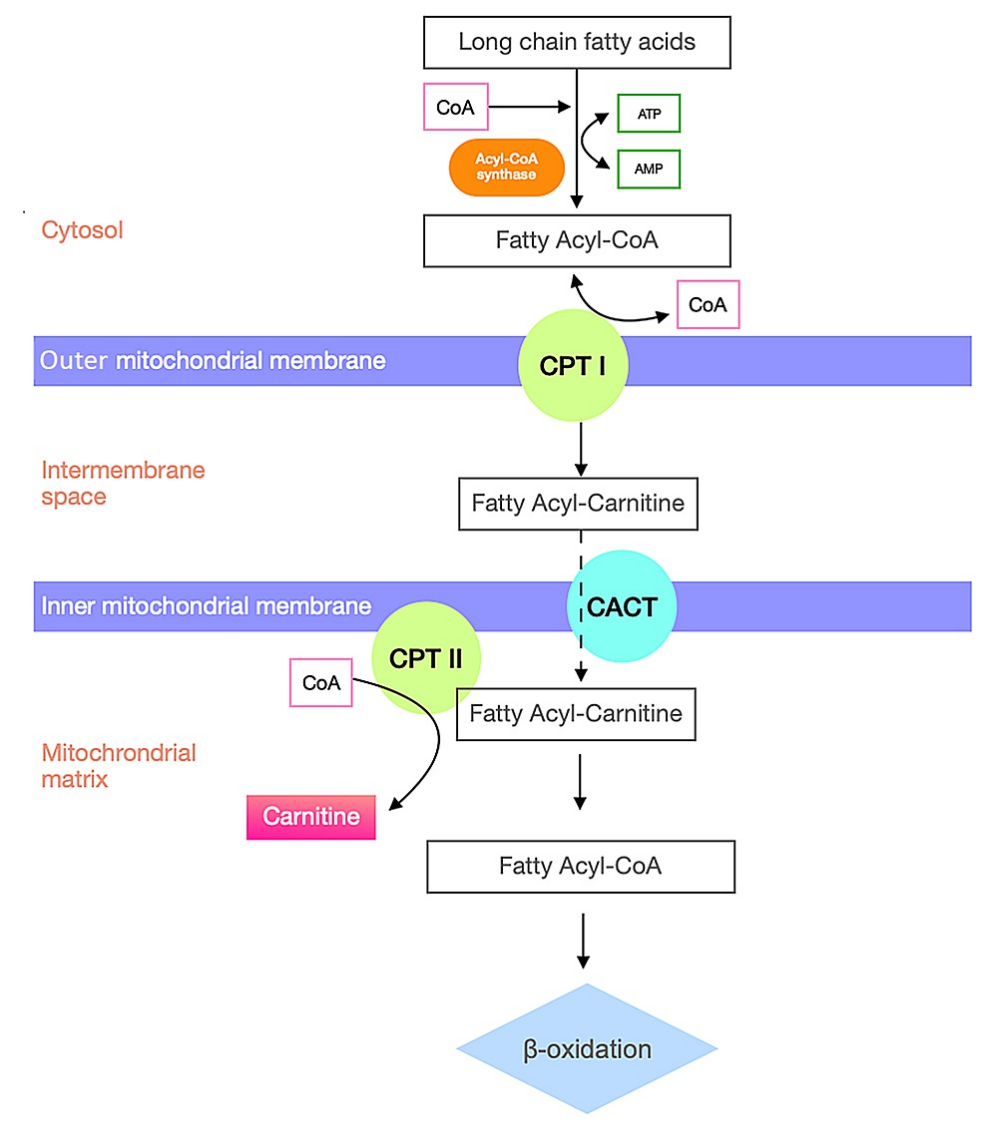
The carnitine shuttle Long-chain fatty acids are converted to fatty acyl-CoAs by the enzyme acyl-CoA synthase, which is subsequently converted to fatty acylcarnitine by the enzyme carnitine palmitoyltransferase I (CPT I) localized in the outer mitochondrial membrane. Fatty acylcarnitine is transported across the inner mitochondrial membrane via carnitine/acylcarnitine translocase (CACT) in exchange for L-carnitine. In the inner mitochondrial membrane, the enzyme carnitine palmitoyltransferase II (CPT II) converts fatty acylcarnitine back to fatty acyl-CoAs and free L-carnitine, which exits the mitochondria and serves as the substrate for CPT I to form more acylcarnitine. These acetyl-CoA carbons then enter the mitochondrial tricarboxylic acid (TCA) cycle.

L-carnitine plays an important role in transporting fatty acids across the mitochondrial membrane for oxidation and the final purpose of energy production, which happens through the carnitine shuttle. An overview of the carnitine shuttle is provided in Figure 3.

Efficacy of Propionyl L-Carnitine

Hiatt et al. showed that there was a statistically significant PWT improvement in three months (P < 0.001) in patients receiving PLC, as compared with those in the placebo group [5]. After six months of treatment, PWT values improved 39% in the PLC group and 14% in the placebo group (P < 0.001), as assessed by a graded treadmill protocol. The mean difference in PWT between the PLC and placebo groups was 87 seconds, equivalent to 255 feet [5]. As perceived by participants, PLC was associated with significant improvements in walking distance (at three and six months) and walking speed (at six months), as compared with placebo (P < 0.05). Participants also recognized a reduction in bodily pain and a transition to a better state of health (P < 0.01) and perhaps better physical function at six months (P = 0.08). Also, there were no significant differences in PLC effects among the two prespecified subgroups (diabetes vs. no diabetes, smokers vs. nonsmokers) [5]. However, a significant treatment-by-country interaction showed that PLC benefits were greater at the Russian sites than at the US sites (P = 0.02). For this, an analysis of variance (ANCOVA) model that included terms for country, treatment, and their interaction demonstrated a statistically significant benefit of PLC (P < 0.01) [5]. Supplementation with oral PLC increases L-carnitine availability for skeletal muscle, which may improve muscle metabolism and vascular endothelial function. Another commonly used medication for IC is cilostazol; it has shown consistent improvements in treadmill performance and quality of life, but patients with heart failure cannot benefit from the use of this drug [5]. No serious adverse events were reported. Thus PLC was both safe and effective in the treatment of claudication [5].

The study of Luo et al. included all patients who received at least one dose of trial medication. The Full Analysis Set (FAS) included all treated patients with at least one post-baseline assessment, and the PerProtocol Set (PPS) included all randomized patients who fulfilled all inclusion/exclusion criteria, completed the trial and follow-up, and took no less than 70% of the trial medication [7]. These two groups, FAS and PPS, were subdivided into intervention and control. In FAS, the PWT of the control group at baseline was 3.55 ± 2.49 minutes, 3.63 ± 2.47 minutes in the third month, and 3.77 ± 2.61 minutes in the fourth month. PWT improved by 0.08 ± 1.22 minutes in the third month and 0.23 ± 1.34 minutes in the fourth month. In PPS, the PWT of the control group at baseline was 3.44 ± 1.96 minutes, 3.52 ± 1.95 minutes in the third month, and 3.67 ± 2.14 minutes in the fourth month. PWT improvement in the third and fourth months was 0.08 ± 1.28 minutes and 0.23 ± 1.40 minutes [7]. In FAS, PWT of the intervention group at baseline, in the third month and fourth months was 3.20 ± 1.63 minutes, 3.94 ± 2.59 minutes, and 4.76 ± 2.95 minutes, respectively; in the third and fourth months, PWT improved by 0.73 ± 1.60 minutes and 1.56 ± 1.88 minutes.

In PPS, the PWT of the intervention group at baseline was 3.17 ± 1.67 minutes, 3.89 ± 2.33 minutes at three months, and 4.81 ± 2.79 minutes at four months. PWT improvement in the third month and the fourth month was 0.72 ± 1.22 minutes and 1.64 ± 1.59 minutes [7]. Improvement in the intervention group was more significant relative to the control group. The intervention group had enhanced 1.33 (0.93 ~ 1.71) minutes more than the control group. Regarding the subjects' general situation at baseline, the variables (age, gender, height, weight, alcohol status, smoking history, and quitting time) did not show any statistically significant differences between the two groups [7]. Also, no significant difference was found between the two groups when evaluating past medical history, history of physical therapy, and the presence of combination therapy (P > 0.05). The incidence of adverse events (AEs) in the intervention group was 30%, higher than that in the control group (25%). The most common AEs were respiratory and gastrointestinal with no permanent damage after drug halting [7]. The incidence of serious adverse events (SAEs) in the intervention group (3.33%) was higher than that in the control group (1.68%). Although PLC may lead to mild AE, overall safety is overweight by its benefits. There were significant differences in PWT between control and treatment groups in both FAS and PPS, which suggests that PLC significantly improved the patients’ ability to walk [7].

In the study of Hiatt et al., PWT had a 75.2% increase in patients randomized to PLC plus exercise, whereas those on placebo had a 64.4% increase. Drug treatment compliance was high (mean of 100.5% on PLC and 98.1% on placebo), but compliance with the exercise program was less (60.2% placebo and 73.7% PLC) [9]. At baseline, 84% of PLC patients and 83% of placebo patients recorded at least 10 hours of ambulatory activity. Monitoring compliance decreased over time, and by the sixth month, ambulatory activity was recorded on only 66% of PLC and 60% of placebo patients [9]. Also, there were no changes in the minutes of ambulatory activity in either group over three and six months. Nonetheless, after six months, the ambulatory activity had increased 1 ± 8% on the placebo group and 0 ± 5% on the treatment group. AEs, which included nausea, diarrhea, bronchitis, and back pain, were described in 70.6% of patients in the treatment group and 68.6% in the placebo group. This study showed no significant benefit of PLC when added to a background of exercise training or benefit in quality of life when compared with exercise training alone in patients with symptomatic PAD [9].

Based on the studies described above, PLC favorably modifies a part of the pathophysiology of claudication supporting their clinical benefit. Since the early 1990s, this topic has been of interest to some researchers that are looking forward to a supplemental treatment for IC symptoms. Side effects and contraindications of the already existing medication for IC are often challenging to physicians due to patients' underlying diseases. The only FDA-approved medical therapies for IC are pentoxifylline and cilostazol, a vasoactive agent that improves the flow of blood by reducing its viscosity and a phosphodiesterase III (PDE3) inhibitor, respectively. Pentoxifylline's most common adverse effects are gastrointestinal related, while cilostazol's most common side effects, despite being a well-tolerated oral medication, include headache, diarrhea, and palpitations [10,11].

Regarding medication interaction, pentoxifylline is the most common one with this property. It may potentiate the effect of antihypertensive agents and can enhance the anti-glycemic action of antidiabetic agents; therefore, a dose reduction of those medications is needed. Also, it can increase the risk of bleeding in patients taking warfarin or antiplatelet agents. Moreover, it increases the plasma levels of theophylline, leading to an excess central nervous stimulation when administered with other xanthine derivatives [10]. Patients contraindicated to take pentoxifylline are those with bleeding disorders or who are at increased risk, patients that exhibit a decreased creatinine clearance (<30 mL/min), patients allergic to xanthine derivatives (caffeine, theophylline, theobromine), patients with acute myocardial infarction or severe coronary disease (due to increased risk of myocardial demand), and those with severe liver disease [10]. Cilostazol is a vasodilator-type agent that generally reduces systemic blood pressure, resulting in a reduction in lower limb perfusion pressure; it can also cause a steal of blood from ischemic regions in which blood vessels are already maximally dilated; therefore, patients with a history of heart failure and patients with a history of ischemic heart disease are contraindicated in the use of cilostazol. Caution is also necessary to individuals with atrial or ventricular ectopy and/or those with atrial fibrillation or flutter [11]. In contrast, PLC supplementation studies showed that AEs were mild and tolerable with no sequelae following its suspension but did not mention any contraindication for its administration [5,7,9].

Future Approach

There are only very few published double-blind, placebo-controlled trials done in 2012 that evaluated the effectiveness of cilostazol/L-carnitine vs. cilostazol/placebo for 180 days [12]. Results demonstrated a trend for efficacy for the combination of these agents in patients with IC. The treatment combination was well tolerated, and the PWT increase from baseline was 1.99 minutes for cilostazol/l-carnitine versus 1.36 minutes for cilostazol/placebo (P = 0.076) [12]. However, exclusion criteria for studied subjects were all cilostazol contraindication mentioned above. All three PLC studies had similar exclusion criteria such as myocardial infarction in the previous six months, uncontrolled hypertension, renal insufficiency, or abnormal liver function [5,7,9], so it is still unclear which medication could be effective for IC symptoms in PAD patients with underlying conditions such as heart failure and ischemic heart disease. The last published clinical trial regarding our topic and outcome of interest was done in 2013 [7]. Therefore, no recent data is available for an update of PLC supplementation in the treatment of IC.

Study Limitations

The present study had several limitations. The analysis was based on a systematic review of three randomized controlled clinical trials that were identified throughout the research and relied upon the availability and accessibility of the publications. The selection of studies for inclusion was based only on one outcome (PWT) and without other combination treatments. The small sample size for all three studies is something that makes it difficult to determine if a particular outcome is a true finding; also, it can produce false-positive results or may overestimate the magnitude of an association.

## Conclusions

In conclusion, study results showed that PLC could effectively improve IC symptoms by increasing maximum walking time and walking distance. The benefit of PLC supplementation may be superior or approaching equivalence to current therapies (cilostazol, naftidrofuryl oxalate, and pentoxifylline). Despite the fact that the three clinical trials used different variables with results that could be affected by the small sample of the study population and influenced by the physical activity of the participants and the subjectivity of the scales used, they showed favorable evidence in the supplementation of PLC for patients with IC in peripheral arterial disease, reducing their symptoms, and improving the walking distance when compared to placebo. While administration of carnitine does not appear to pose any safety concerns, long-term evidence is not currently available. Further clinical trials are needed to investigate the effectiveness of the supplemental treatment for improving exercise performance in patients with IC symptoms. Also, new severity scales, vigilance tests, and patient-reported outcomes are needed.
